# Influence of nanoparticles of platinum on chicken embryo development and brain morphology

**DOI:** 10.1186/1556-276X-8-251

**Published:** 2013-05-24

**Authors:** Marta Prasek, Ewa Sawosz, Slawomir Jaworski, Marta Grodzik, Teresa Ostaszewska, Maciej Kamaszewski, Mateusz Wierzbicki, Andre Chwalibog

**Affiliations:** 1 Faculty of Animal Science, Division of Biotechnology and Biochemistry of Nutrition, Warsaw University of Life Science, Warsaw 02-786, Poland; 2 Faculty of Animal Science, Division of Ichthyobiology and Fisheries, Warsaw University of Life Science, Warsaw 02-786, Poland; 3 Department of Veterinary Clinical and Animal Sciences, University of Copenhagen, Groennegaardsvej 3, Frederiksberg 1870, Denmark

**Keywords:** Platinum nanoparticles, Chicken embryo, Toxicity, Neurotoxicity, Brain morphology, Cancer therapy

## Abstract

Platinum nanoparticles (NP-Pt) are noble metal nanoparticles with unique physiochemical properties that have recently elicited much interest in medical research. However, we still know little about their toxicity and influence on general health. We investigated effects of NP-Pt on the growth and development of the chicken embryo model with emphasis on brain tissue micro- and ultrastructure. The embryos were administered solutions of NP-Pt injected *in ovo* at concentrations from 1 to 20 μg/ml. The results demonstrate that NP-Pt did not affect the growth and development of the embryos; however, they induced apoptosis and decreased the number of proliferating cells in the brain tissue. These preliminary results indicate that properties of NP-Pt might be utilized in brain cancer therapy, but potential toxic side effects must be elucidated in extensive follow-up research.

## Background

Platinum (Pt) is a noble metal with unique physiological and chemical properties widely used in chemistry, physics, biology, and medicine. Regarding the biological activities of Pt, it is known that Pt compounds have the ability to arrest the cell cycle [1,2] and cause DNA strand breaks. The DNA damage is caused by Pt ions, which attach to N7 sites of DNA guanine bases and, after hydrolysis of Pt-Cl bonds, form adducts with the DNA double helix [2,3]. These properties of Pt are exploited in cancer therapy in the form of antineoplastic drugs to treat different types of cancer such as head, neck, brain [4], testicular, bladder, ovarian, or uterine cervix carcinomas [5]. However, toxic side effects of Pt-based drugs are major drawbacks in cancer therapy [6,7].

Nanotechnology has introduced possibilities for using alternate forms of elements - nanoparticles. Nanoparticles have unique physiochemical features because of their small size (<100 nm), large surface-to-mass ratio, exceptional quantum characteristics [8], and consequently unique biological properties. Smaller nanoparticles can move across cellular and also nuclear membranes and are able to penetrate cells and intracellular structures, and target defined points within the body [9,10]. Platinum nanoparticles (NP-Pt) have recently elicited much interest because of their physicochemical properties such as catalytic activity and high reactivity [11]. NP-Pt, as metal structures (Pt^0^), differ significantly from platinum salts and have quite different chemical properties when administered to an organism. They are a very limited source of ions, and consequently, the process of forming platinum salts is very slow and restricted. However, the solubility and, consequently, the bioavailability of NP-Pt depend on their size [12]. Although it has been demonstrated that small doses of NP-Pt have negligible toxic effects on chicken and zebra fish embryos [13], they might impinge the cell structures [12].

It has been demonstrated that hadron cancer therapy can be amplified by simultaneous application of NP-Pt, resulting in the production of hydroxyl radicals causing lethal DNA damage by double-strand breaks [14]. Furthermore, DNA damage could also be induced by the attack of OH groups linked with NP-Pt on DNA phosphate groups [2]. NP-Pt can also cause cell cycle arrest and induction of apoptosis through the release of Pt^2+^ ions from the nanoparticles as a result of H_2_O_2_ generation due to the low pH in endosomes [1]. It was also demonstrated that DNA double-strand breaks are caused by Pt^2+^ ions formed during the incubation of NP-Pt with cancer cells [15]. However, the consequences of introducing NP-Pt into an organism are still not well documented, especially when even very small amounts of nanoparticles or released ions may overcome the blood–brain barrier (BBB), enter the brain tissue, and affect the BBB and brain function. It has also been reported that various types of nanoparticles, in different sizes from 20 to 300 nm and produced from different materials, may cause cell death by apoptosis in the brain tissue [16].

In the present study, we hypothesized that NP-Pt may affect the growth and development of embryos and, furthermore, can cross the BBB and penetrate the brain tissue, affecting brain morphology. Consequently, the objective of this preliminary work was to investigate the effects of NP-Pt on embryo growth and development with an emphasis on brain morphology, concerning their potential applicability in brain cancer therapy.

## Methods

### Nanoparticles

**Figure 1:**
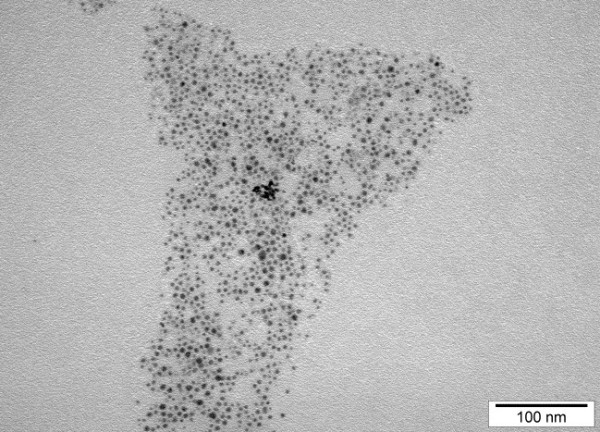
**TEM image of platinum nanoparticles.** Bar scale 100 nm.

Hydrocolloids of NP-Pt were obtained from Nano-Tech Polska (Warsaw, Poland). They were produced by a patented electric nonexplosive method [17] from high purity metal (99.9999%) and high purity demineralized water. The shape and size of the nanoparticles were inspected by transmission electron microscopy (TEM) using a JEOL JEM-1220 TE microscope at 80 KeV (JEOL Ltd., Tokyo, Japan), with a Morada 11 megapixel camera (Olympus Corporation, Tokyo, Japan) (Figure 1). The diameters of the Pt particles ranged from 2 to 19 nm. A sample of Pt for TEM was prepared by placing droplets of the hydrocolloids onto Formvar-coated copper grids (Agar Scientific Ltd., Stansted, UK). Immediately after drying the droplets in dry air, the grids were inserted into the TE microscope (Figure 1). The zeta potential of the nanoparticle hydrocolloids was measured by electrophoretic light-scattering method, using a Zetasizer Nano-ZS90 (Malvern, Worcestershire, UK). Each sample was measured after 120 s of stabilization at 25°C in 20 replicates. The mean zeta potential of the Pt nanoparticles was −9.6 mV.

### Embryo model

Based on Polish law Article 2 of the act dated 21 January 2005 concerning the experiments on animals (journal of law is dated 24 February 2005), there is no need to submit an application to the local ethics committee for issuing an opinion about studies where the chicken embryo is used. According to this act, chicken embryo is not definite as the animal. Fertilized eggs (*n* = 150; 56 ± 2.2 g) from hens of the Ross line were obtained from a commercial hatchery and stored at 12°C for 4 days. After 4 days, the eggs were weighed and randomly divided into six groups (*n* = 25 eggs per group). The control group was not treated, while the other groups were treated with 1, 5, 10, 15, or 20 μg/ml of NP-Pt solutions. The experimental solutions were given *in ovo* by injection into the albumen (at two-thirds of the egg's height from the blunt end) using a sterile 1-ml insulin syringe. Injection consisted of 0.3-ml NP-Pt hydrocolloid. The injection holes were sterilized, and the eggs were then incubated at 37.5°C and 60% humidity and were turned once per hour for 19 days.

At day 20 of incubation, the embryos were sacrificed by decapitation. Embryos and organs (brain, heart, liver, spleen, bursa of Fabricius) were weighed and evaluated by Hamburger and Hamilton [18] (HH) standards.

### Biochemical indices

Blood serum samples were collected from the jugular vein on the 20th day of incubation. The samples were centrifuged at 3,000 rpm for 15 min (Sorvall ST 16, Thermo Fisher Scientific, Waltham, MA, USA), and concentrations of alanine aminotransferase (ALT), asparagine aminotransferase, lactate dehydrogenase, alkaline phosphatase (ALP), glucose level, and blood urea nitrogen were measured in the blood serum. Biochemistry markers were examined using a dry chemistry equipment Vitros DT 60 II (Johnston and Johnston, New Brunswick, NJ, USA).

### Brain morphology: examination of brain tissue microstructure

Chicken brains (*n* = 12), three from the control group and nine from groups treated with 1, 10, and 20 μg/ml of NP-Pt solutions, were sampled and fixed in 10% buffered formalin (pH 7.2). Fixed samples were dehydrated in a graded series of ethanols, embedded in Paraplast, and cut into 5-μm sections using a microtome (Leica RM 2265, Leica, Nussloch, Germany). The morphology of the chicken brains was examined using hematoxylin-eosin staining. Proliferating cells were identified via immunohistochemistry using antibodies directed against proliferating cell nuclear antigen (PCNA) [19]. Apoptotic cells were detected using rabbit polyclonal anti-caspase-3 antibody (C8487, Sigma-Aldrich Corporation, St. Louis, MO, USA). Sections for this purpose were incubated for 1 h with the rabbit polyclonal anti-caspase-3 antibody at room temperature and were visualized with Dako EnVision^+^System-HRP (Dako K 4010, Dako A/S, Glostrup, Denmark), while further procedures were identical as for PCNA detection. The proliferation and apoptosis levels were expressed as the number of PCNA-positive cells and caspase-3-positive cells in the chicken brain cortex, respectively (the area counted was 3,500 μm^2^).

Morphological observations and morphometric measurements (number of PCNA-positive cells, caspase-3-positive cells) were done using a Nikon ECLIPSE 90i microscope (at ×400 magnification) connected to a digital camera, Nikon DS5-U1, and a computer image analysis system, NIS-Elements AR (Nikon Corporation, Tokyo, Japan).

### Examination of brain tissue ultrastructure

Brain tissue morphology was examined by TEM. The tissues were fixed for TEM in fixative consisting of 1% glutaraldehyde in PBS at pH 7.2. After fixation, the tissues were post-fixed in 1% osmium tetroxide and dehydrated in a graded series of ethanols. The tissues were embedded in a mixture of Araldite and Epon. Ultrathin sections (100 nm) were cut on an ultramicrotome (EM UC6, Leica). The samples were viewed using a JEM-1220 TE microscope at 80 KeV (JEOL Ltd.), with a Morada 11 megapixel camera (Olympus Corporation).

### Statistical analysis

Data analysis was carried out by monofactorial analysis of variance, and the differences between groups were tested by multiple range Duncan test using Statistica version 10.0 (StatSoft, Tulsa, OK, USA). Differences with *P* < 0.05 were considered significant.

## Results and discussion

### Results

#### Growth and development

Embryo visualization did not show any genetic defects among the groups. Furthermore, comparison with HH standards showed that all embryos had developed normally. Survival, body weight, and weight of the brain, heart, spleen, and bursa of Fabricius were not significantly different between all the groups (Table 1). However, the weight of the liver was significantly different in some NP-Pt groups compared to the control group. None of the biochemical indices measured in the blood sera of the embryos showed significant effects of the treatments (Table 2).

**Table 1:** Survival, body weight, and selected organ weight in control and groups treated with different NP-Pt concentrations

	**Control**	**1.0 μg/ml**	**5.0 μg/ml**	**10.0 μg/ml**	**15.0 μg/ml**	**20.0 μg/ml**	**SEM**	***P*value**
Survival	25	20	19	20	21	21	0.4837	0.1152
Body	50.77	53.97	52.97	53.15	54.30	52.00	5.043	0.2510
Brain	0.434	0.453	0.328	0.474	0.471	0.455	0.0564	0.6855
Heart	0.165	0.146	0.152	0.154	0.145	0.128	0.0475	0.0806
Liver	0.559 b	0.434 a	0.475 a	0.52 ab	0.495 a	0.516 ab	0.1645	0.0405^*^
Spleen	0.013	0.010	0.015	0.012	0.010	0.009	0.0122	0.5891
Bursa of Fabricius	0.030	0.025	0.028	0.029	0.028	0.030	0.2559	0.9815

Chicken embryo survival (number of embryos), body weight (g), and weight of selected organs (g/100 g body weight) in the control group and in groups treated with different concentrations of platinum nanoparticles (1 to 20 μg/ml). *SEM* standard error of the mean. Means with different letters differ significantly; *P < 0.05.

**Table 2:** Activities of biochemical indices in the control and in groups treated with different NP-Pt concentrations

**Biochemical indices**	**Reference values**^**a**^	**Control**	**1.0 μg/ml**	**10.0 μg/ml**	**20.0 μg/ml**	**SEM**	***P*value**
Asparagine aminotransferase (U/l)	90 to 226	193.1	214.2	183.4	170.1	15.35	0.4845
Alanine aminotransferase (U/l)	9 to 14	11.78	8.53	17.00	18.25	4.399	0.2182
Lactate dehydrogenase (U/l)	1,100 to 5,275	1,135	1,121	778	1,026	83.6	0.2073
Alkaline phosphatase (U/l)	3,780 to 14,800	6,300	4,800	4,030	7,033	47.8	0.0712
Blood urea nitrogen (mg/Dl)	7.0 to 17.1	5.7	8.0	7.5	8.0	0.41	0.1272
Glucose level (mg/Dl)	110 to 306	219	213	169	203	8.2	0.1269

*SEM* standard error of the mean. ^a^Reference values of biochemical indices for poultry [20].

#### Brain morphology: examination of brain tissue microstructure

**Figure 2:**
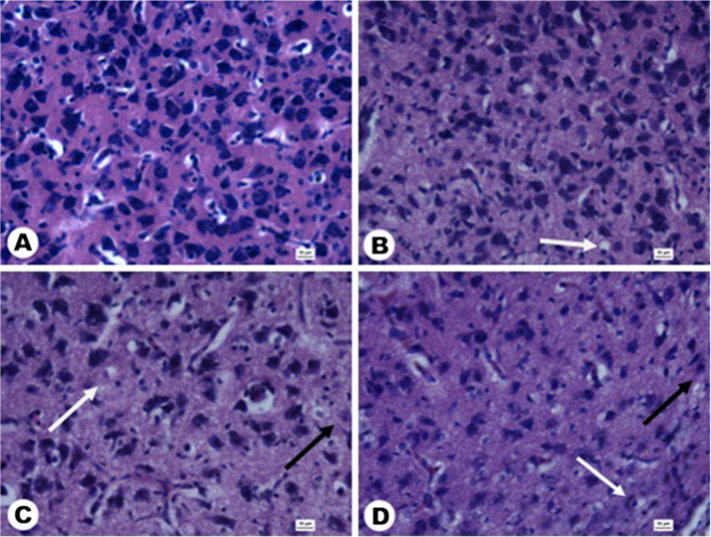
**Cross sections through the granular layer of the cerebral cortex stained with hematoxylin and eosin.** (**A**) Control, (**B**) 1 μg/ml, (**C**) 10 μg/ml, (**D**) 20 μg/ml. Black arrows, astrocytosis; white arrows, neuronal loss**.** Scale bars 10 μm.

Cell numbers in the brain cortex (area counted 3,500 μm^2^) were not significantly different between the groups (Table 3). However, histological evaluation of brain morphology revealed pathological changes in the brain structure in embryos treated with NP-Pt, showing a moderate degradation of the cerebellar molecular layer, neuronal loss in the cerebellum cortex, and astrocytosis (Figure 2).

**Table 3:** Numbers of cells in the brain cortex in the control and in groups treated with different NP-Pt concentrations

	**Control**	**1.0 μg/ml**	**10.0 μg/ml**	**20.0 μg/ml**	**SEM**	***P*value**
Number of cells	613	583	600	697	6.5	0.448

#### Examination of brain tissue ultrastructure

**Figure 3:**
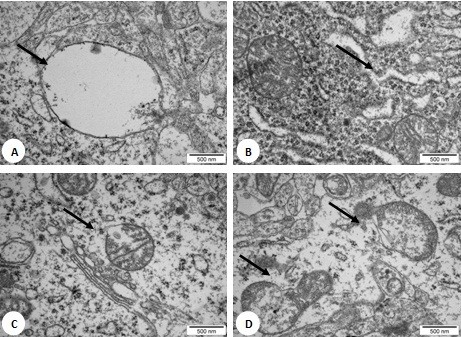
**TEM images of brain tissue after treatment with platinum nanoparticles.** Concentration of NP-Pt was at 20 ppm. Arrows signify (**A**) vacuoles, (**B**) degradation of endoplasmic reticulum, and (**C, D**) degradation of the mitochondria. Scale bars 500 nm.

TEM examination of brain tissue morphology showed no abnormalities in the control group. However, in embryos treated with NP-Pt, degradation of the mitochondria, rounded nuclei with dispersed chromatin, and vacuoles in the cytoplasm were seen (Figure 3).

**Figure 4:**
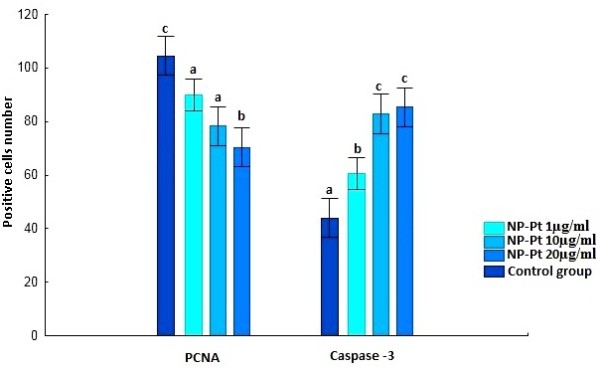
**Numbers of caspase-3-positive cells and PCNA positive nuclei (counting area = 3,500 μm**^**2**^**).** Error bars indicate standard error of the mean. Bars with different superscripts differ significantly (*P* < 0.05).

**Figure 5:**
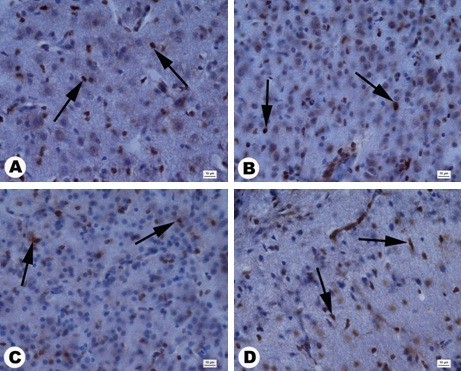
**Cross sections of a granular layer in the cerebral cortex by PCNA staining.** (**A**) Control, (**B**) 1 ppm, (**C**) 10 ppm, and (**D**) 20 ppm. PCNA-positive nuclei (arrows). Scale bars 10 μm.

**Figure 6:**
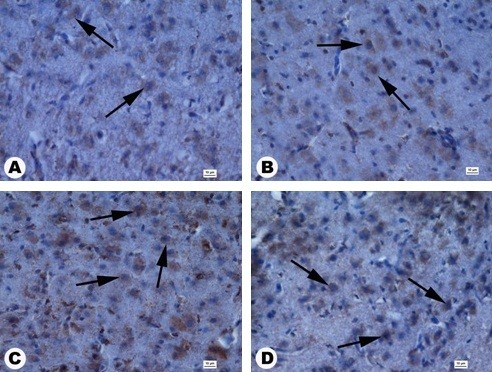
**Cross sections of a granular layer in the cerebral cortex by anti-caspase-3 staining.** (**A**) Control, (**B**) 1 μg/ml, (**C**) 10 μg/ml, (**D**) 20 μg/ml. Anti-caspase-3-positive cells (arrows). Scale bars 10 μm.

Immunohistochemical measurements showed that the number of PCNA-positive nuclei significantly decreased after *in ovo* injection of NP-Pt solutions, attaining the lowest value in the 20-μg/ml group (Figure 4). Immunodetection of PCNA-positive nuclei by immunohistochemical methods was carried out in cross sections of the granular layer of the cerebellar cortex. PCNA-positive nuclei were brown, and PCNA-negative nuclei were blue (Figure 5). Immunohistochemical measurements showed the numbers of caspase-3-positive cells significantly increased in the NP-Pt groups compared to those in the control group (Figure 4). The greatest increase was observed in the group receiving 20 μg/ml of NP-Pt. Cross sections of the granular layer of cerebral cortex were also immunostained with the caspase-3 antibody. Caspase-3-positive cells showed brown cytoplasm, while the cytoplasm of caspase-3-negative cells was blue (Figure 6).

## Discussion

In the present work, we studied the effects of different concentrations of platinum nanoparticle hydrocolloids administered to chicken embryos on their growth and development as well as on the morphological and molecular status of the brain at the end of embryogenesis. The chicken embryo is a very useful experimental model, developing without influence of the maternal organism and allowing very fast and precise assessments of toxicity [21,22]. Moreover, NP-Pt were administered at the beginning of embryogenesis, when, consequently, nanoparticles could potentially penetrate the entire organism, including brain precursor cells, differentiated cells, and brain structures, both before and after the appearance of the BBB [7].

Our studies demonstrated that NP-Pt injected into eggs at concentrations of 1, 5, 10, 15, and 20 μg/ml did not influence the growth and development of the chicken embryos. Their survival as well as examination of their morphology according to HH standards of chicken embryo development did not differ between the control and NP-Pt groups. No overt abnormalities that could indicate mutagenic effects of NP-Pt were observed. These results are in agreement with a recent investigation demonstrating no toxic effects of NP-Pt on the growth and development of *Danio rerio* embryo [13]. Furthermore, they are in agreement with our own previous studies regarding the effects of nanoparticles of silver, silver/palladium alloy, and gold, showing no harmful effects on growth and development of embryos when the nanoparticles were used at concentrations below 100 μg/ml [23-27]. In contrast to NP-Pt, platinum-based drugs such as *cis*-dichlorodiammineplatinum (II) (cisplatin) do show toxic effects on the development and mortality of rat embryos [28]. Platinum compounds also have toxic effects on mouse embryo development during organogenesis and histogenesis [29].

In our experiment, body weight and the weights of selected organs in the chicken embryos were not significantly affected by NP-Pt injection; however, liver weight was generally lower in the NP-Pt groups compared to the control group, which might indicate some harmful effects of NP-Pt. Subsequently, we measured the activities of hepatic enzymes in blood serum (ALT, AST, and ALP) as markers of the functional and morphological state of the liver [5], but these indices were not affected by NP-Pt. Consequently, our preliminary observations regarding growth and development suggest that NP-Pt do not seem to be harmful when evaluated at the whole body and organ level; however, potential subclinical changes might occur at the tissue and molecular levels.

The chicken embryo is a suitable model to study neurotoxicity because the BBB is fully developed and functioning after 15 days of incubation [7]. The key role of the BBB is protecting the brain from toxic substances. On the other hand, the blocking role of the BBB is problematic because drugs used to treat many diseases of the central nervous system are unable to cross this highly specific barrier [30]. Application of NP-Pt at the beginning of embryogenesis makes it possible for NP-Pt to penetrate different tissues, including brain precursor cells and structures. Moreover, enclosed and separated from the mother and environment, the organism has no possibilities to remove the nanoparticles from the egg, and consequently, the embryos were permanently exposed to PN-Pt during 20 days of embryogenesis (before and after BBB occurrence).

The present results demonstrated that PN-Pt did not cause any difference in brain weight evaluated at the end of embryogenesis. Histological assessment of the brain structure revealed some minor pathological changes, but the number of brain cortex cells was not affected. However, more detailed examination of the brain tissue ultrastructure indicated some neurotoxic symptoms from NP-Pt treatment, including deformation and degradation of the mitochondria. Similar results were obtained for cisplatin, showing mitochondrial and nuclear DNA damage in the dorsal root ganglia [31]. Thus, not only platinum salts but also NP-Pt, via mitochondrial disruption, may lead to mitochondria-dependent apoptosis. Although almost half the neuronal cells die by apoptosis during normal brain development, this physiological process may be enhanced under toxic conditions [32]. However, the stimulation of mechanism of apoptosis within tumor cells is considered a highly advanced cancer therapy [33] and, in this respect, NP-Pt can enhance the apoptosis of cancer cells.

Cytochrome c released from the mitochondria into the cytosol is one of the first steps in the mitochondrial apoptotic pathway. Cytochrome c and ATP are bound to the apoptotic protease-activating factor-1 [34]. The merger of these two structures creates an apoptosome and activates caspase-9. Active caspase-9 is responsible for the activation of the executioners, caspase-3 and caspase-7 [32,35]. We examined the activity of caspase-3 to detect apoptosis. Our results showed an increasing level of caspase-3-positive cells in chicken brain samples from groups treated with NP-Pt. These results agree with studies suggesting that platinum-based drugs trigger DNA damage, which induces apoptosis with the activation of caspase-3 [36,37].

Opposing apoptosis is the process of cell proliferation, and thus, the interaction between apoptosis and proliferation, observed after platinum-based drug treatment, is a key factor in cancer therapy [38]. To examine the status of proliferation after NP-Pt treatment, we also identified the level of PCNA-positive nuclei in the brain tissue. The immunohistochemical analyses showed a decline in PCNA-positive nuclei in the NP-Pt groups. PCNA is a key factor in the replication of genetic material and is involved in the cell cycle and proliferation processes [39]. This may indicate that NP-Pt analogs to platinum-based drugs, where Pt exists in cationic form, activate apoptosis and at the same time suppress proliferation. However, the toxic side effects of NP-Pt seem to be much smaller than those caused by platinum-based drugs containing ionic Pt. This may suggest that NP-Pt could be used in cancer therapy instead of ionic Pt, especially for brain cancer, because the particles can pass the BBB and reach the tumor tissue in the brain.

## Conclusions

Platinum nanoparticles administered to chicken embryos at the beginning of embryogenesis at concentrations of 1 to 20 μg/ml did not affect the growth and development of the embryos. Examination of neurotoxicity after NP-Pt treatment showed no changes in the number of cells in the brain cortex; however, analyses of brain tissue ultrastructure revealed mitochondria degradation. NP-Pt activated apoptosis as well as decreased the rate of proliferation of the brain cells. These preliminary results indicate that properties of NP-Pt might be utilized for brain cancer therapy, but potential toxic side effects must be elucidated in extensive follow-up research.

## Abbreviations

ALT: Alanine aminotransferase; ALP: Alkaline phosphatase; BBB: Blood–brain barrier; cisplatin: *Cis*-dichlorodiammineplatinum (II); HH standard: Hamburger and Hamilton standard; NP-Pt: platinum nanoparticles; PCNA: Proliferating cell nuclear antigen; TEM: Transmission electron microscopy.

## Competing interests

The authors declare that they have no competing interests.

## Authors’ contributions

MP carried out *in ovo* studies and drafted the manuscript. ES conceived the study and helped draft the manuscript. SJ participated in the analysis of biochemical indices. TO participated in the histological studies and helped draft the manuscript. MK participated in the immunohistological studies. MG participated in the design the experiment. MW participated in the statistical analysis. AC participated in the design and coordination and helped draft the manuscript. All authors read and approved the final manuscript.

## Authors’ information

MP is a PhD student at the Warsaw University of Life Sciences (WULS). ES has PhD and DSc degrees and is a professor and head of a department at WULS. SJ is a PhD student at WULS. MG has PhD and postdoctorate degrees at WULS. TO has PhD and DSc degrees and is a professor and head of a department at WULS. MK has PhD and postdoctorate degrees at WULS. MW is a PhD student, and AC has a DSc degree and is a professor and head of a division at the University of Copenhagen (UC).
